# Beneficial effect of combined treatment with octreotide and pasireotide in PCK rats, an orthologous model of human autosomal recessive polycystic kidney disease

**DOI:** 10.1371/journal.pone.0177934

**Published:** 2017-05-18

**Authors:** Masanori Kugita, Kazuhiro Nishii, Tamio Yamaguchi, Atsushi Suzuki, Yukio Yuzawa, Shigeo Horie, Eiji Higashihara, Shizuko Nagao

**Affiliations:** 1 Education and Research Center of Animal Models for Human Diseases, Fujita Health University, Toyoake, Aichi, Japan; 2 Faculty of Rehabilitation, School of Health Sciences, Fujita Health University, Toyoake, Aichi, Japan; 3 Department of Clinical Nutrition, Faculty of Health Science, Suzuka University of Medical Science, Suzuka, Mie, Japan; 4 Division of Endocrinology and Metabolism, Department of Internal Medicine, Fujita Health University, Toyoake, Aichi, Japan; 5 Department of Nephrology, Fujita Health University School of Medicine, Toyoake, Aichi, Japan; 6 Department of Urology, Juntendo University School of Medicine, Bunkyo-ku, Tokyo, Japan; 7 Department of Urology, Kyorin University School of Medicine, Mitaka, Tokyo, Japan; Istituto Di Ricerche Farmacologiche Mario Negri, ITALY

## Abstract

Increased intracellular cyclic AMP (cAMP) in renal tubular epithelia accelerates the progression of polycystic kidney disease (PKD). Thus, decreasing cAMP levels by an adenylyl cyclase inhibitory G protein activator is considered to be an effective approach in ameliorating PKD. In fact, pasireotide (PAS) was effective in reducing disease progression in animal models of PKD. However, hyperglycemia caused by the administration of PAS is an adverse effect in its clinical use. Whereas, co-administration of octreotide (OCT) with PAS did not increase serum glucose in normal rats. In the current study, we examined the efficacy of combined treatment with OCT and PAS in PCK rats, an autosomal recessive PKD model. Four-week-old PCK males were treated with the long-acting release type of OCT, PAS, or a combination of both (OCT/PAS) for 12 weeks. After termination, serum and renal tissue were used for analyses. Kidney weight, kidney weight per body weight, renal cyst area, renal Ki67 expression, and serum urea nitrogen were significantly decreased either in the PAS or OCT/PAS group, compared with vehicle. Renal tissue cAMP content was significantly decreased by PAS or OCT/PAS treatment, but not OCT, compared with vehicle. As a marker of cellular mTOR signaling activity, renal phospho-S6 kinase expression was significantly decreased by OCT/PAS treatment compared with vehicle, OCT, or PAS. Serum glucose was significantly increased by PAS administration, whereas no difference was shown between vehicle and OCT/PAS, possibly because serum glucagon was decreased either by the treatment of OCT alone or co-application of OCT/PAS. In conclusion, since serum glucose levels are increased by the use of PAS, its combination with OCT may reduce the risk of hyperglycemia associated with PAS monotherapy against PKD progression.

## Introduction

Human polycystic kidney disease (PKD) is characterized by the progressive formation of numerous fluid-filled cysts in the kidney, and in most cases, hepatic fibrocystic disorders occur. Cyst development is caused by the stimulation of cell proliferation in the renal tubule and hepatic bile duct epithelia with fluid secretion into the lumen. There are two major types of PKD: autosomal dominant PKD (ADPKD) and autosomal recessive PKD (ARPKD). ADPKD is caused by *PKD1* or *PKD2* gene mutation and its incidence in humans is 5:10,000 [1]. Most cases are diagnosed after adulthood, and end-stage renal disease occurs in 50% of patients [2]. ARPKD is a juvenile-type cystic disease caused by mutation of the *PKHD1* gene with an incidence of 1:20,000 [3]. Renal cysts are formed from collecting ducts with fibrotic tissue. Liver cysts are formed from bile ducts connected to the biliary tree embedded in fibrotic tissue, often complicated with ductal plate malformation caused by congenital hepatic fibrosis [4]. In these PKDs, the stimulated cell proliferation of the renal tubule and hepatic bile duct epithelia is caused by increased levels of intracellular adenosine 3′,5′-cyclic monophosphate (cAMP), a second messenger that upregulates B-Raf/MEK/ERK signaling in animal models and patients [5, 6]. The intracellular concentration of cAMP is regulated through receptors bound to G proteins, the stimulatory G protein (Gs) or inhibitory G protein (Gi) of adenylyl cyclase, which are activated by extracellular signals from certain hormones. Therefore, investigators have tried to suppress the action of these hormones either by inhibiting Gs or stimulating Gi activity in animal models and PKD patients to ameliorate disease progression [7–12]. An antidiuretic hormone, arginine vasopressin (AVP), is known to increase intracellular cAMP concentration in several tissues including the kidney and liver. Tolvaptan, an AVP V2 receptor antagonist that inhibits Gs activity stimulated by AVP, reduces cAMP production in renal epithelia and ameliorates disease progression in animal models and human clinical trials, as the first available medicine against PKD [7–9]. Further, increased water consumption suppresses the secretion of AVP and ameliorates PKD progression in an animal model [13]. These findings indicate that reducing cAMP production is potentially effective in this disorder.

Octreotide (OCT) is a somatostatin analog that binds to the somatostatin receptor (SSTR), mainly subtypes 2, 3, and 5, increases Gi activity, and reduces intracellular cAMP production [14]. OCT is expected to be a new candidate therapeutic agent for PKD patients. Clinical studies have been reported by several investigators [12, 15–18]. Most recently, the ALADIN (a long-acting somatostatin on disease progression in nephropathy) study was performed [12], and a long-acting release (LAR) type of OCT reduced the volume of the cysts and total kidney after treatment for 1 year, but not after 3 years. Further, OCT-LAR decreased the glomerular filtration rate after treatment for 3 years, but not after 1 year. Therefore, the ameliorative or deteriorative effects of OCT-LAR treatment in PKD patients have not been evaluated clearly. Masyuk et al. reported that OCT reduces disease progression in the kidney and liver of the PCK rat strain, a human orthologous rodent model of ARPKD, with a reduction of intracellular cAMP concentration in cholangiocytes [10]. Furthermore, they reported that pasireotide (PAS), another somatostatin analog that binds to SSTR 1, 2, 3, and 5 and reduces cAMP synthesis [14], is more effective than OCT in reducing renal and hepatic disease progression in this rat model [11]. The different potency of these two somatostatin analogs may arise from their targeted receptor subtype; OCT upregulates only SSTR2, whereas PAS upregulates either SSTR1 or SSTR2 [11].

However, PAS causes hyperglycemia in the clinical setting [19]; whereas, co-administration of OCT and PAS does not raise serum glucose levels in rats [20]. Hence, in the present study, we determined the effect of co-treatment with OCT and PAS in PCK rats to assess whether this combination causes an ameliorative effect without leading to hyperglycemia.

## Materials and methods

### Animals and study design

The PCK rat strain used in the current study was originally derived from a Sprague-Dawley colony in Charles River Japan. The descendants have been bred and maintained at the Education and Research Center of Animal Models for Human Diseases, Fujita Health University. In this strain, by a splicing mutation with subsequent skipping of exon 36 and a frameshift in the human orthologous *Pkhd1* gene, kidney and liver disorders occur [21]. These are characterized by renal cysts originated from collecting ducts, and congenital hepatic fibrosis complicated with biliary cysts [22]. In the current study, the rats were allowed free access to laboratory chow and water [23, 24]. The LAR type of OCT and PAS, and vehicle were obtained from Novartis (Basel, Switzerland).

Male PCK rats (*n* = 24) were assigned randomly to 1 of 4 groups (*n* = 6 per group): treatment by subcutaneous injection every 4 weeks treatment with 8 mg/kg OCT-LAR alone, 8 mg/kg PAS-LAR alone, co-application of 8 mg/kg OCT and 8 mg/kg PAS, or vehicle (microparticles liquid; CONT) from 4 to 16 weeks of age. The vehicle contains copolymer microparticles with polylactic-co-glycolic acid (PLGA), obtained from Novartis. In 4- and 15-week-old conscious rats, heart rate (HR), diastolic blood pressure (DBP), and systolic blood pressure (SBP) were determined using a tail-cuff sphygmomanometer (BP98A; Softron Co., Ltd., Tokyo, Japan) [23]. Twenty-four-hour urine volume and food consumption were measured using metabolic cages after 15.5 weeks of age [13]. After body weight measurement, the animals were anesthetized with sodium pentobarbital (Schering-Plough Corp., Kenilworth, NJ, USA) at 16 weeks of age, and the kidneys and liver were removed rapidly, causing lethal exsanguination. Total wet kidney weight and wet liver weight were measured, and blood samples were collected for measurements of serum urea nitrogen (SUN), aspartate amino transferase (AST), alanine aminotransferase (ALT), insulin-like growth factor-1 (IGF-1), glucose, insulin, glucagon, and cortisol.

To extract proteins, the left kidney was homogenized in ice-cold Triton lysis buffer [23, 24]. For immunohistochemistry, the right kidney and a part of the right medial liver lobe were immersed in 4% paraformaldehyde overnight at 4°C, displaced in phosphate-buffered saline without Mg^2+^ and Ca^2+^ (PBS^-^), dehydrated by ethanol, dealcoholized by xylene, embedded in paraffin, and sectioned for immunohistochemistry staining.

### Ethics statement

The rats were handled ethically according to the Regulations for the Management of Laboratory Animals at Fujita Health University. The experimental protocol for the ethical use of these animals was approved by the Animal Care and Use Committee at Fujita Health University (Permit No.: M2834).

### Measurement of serum levels of SUN, IGF-1, glucose, insulin, glucagon, cortisol, AST, ALT, and renal tissue cAMP content

Serum biochemical parameters were analyzed by the following methods. SUN measurements were determined by a colorimetric assay using a urease-indophenol method (Wako Pure Chemicals, Osaka, Japan). IGF-1 and glucose measurements were analyzed with an enzyme-linked immunosorbent assay (ELISA; MG100; R&D Systems, Minneapolis, MN, USA) and a colorimetric assay by the mutarotase-glucose oxidase method (Glucose CII-test; Wako), respectively. Insulin and glucagon were measured using ELISA kits (AKAIN-010T; Shibayagi, Gunma, Japan, and Wako, respectively). Cortisol was analyzed by an ELISA kit (KGE008; R&D Systems). AST and ALT were measured by the pyruvate oxidase-N-ethyl-N-(2-hydroxy-3-sulfopro-pyl)-m-toluidine method with a commercial kit (Transaminase CII-test; Wako). To measure renal tissue cAMP content, kidneys were prepared by homogenizing with HCl. Extracted cAMP levels were analyzed by using a cAMP radioimmunoassay kit (Yamasa, Chiba, Japan), and were corrected by protein concentration.

All biochemical parameters were measured according to the manufacturers’ instructions and are expressed as the mean ± standard deviation (SD) (*n* = 6 rats per treatment group).

### Cyst area and fibrosis index

Cystic area was determined using 10 random fields (×100 magnification) of hematoxylin and eosin-stained kidney and liver sections from PCK rats. Fibrosis index was detected from 10 random fields (×100 magnification) of picrosirius red-stained sections of the liver. The picrosirius red-stained sections were also used to observe collagen fibers by polarized light microscopy [23, 24]. Cyst area and fibrosis index were measured and calculated by a naive observer using LUZEX AP software (NIRECO Co., Ltd., Tokyo, Japan) and are expressed as mean ± SD values.

### Western blot analysis

From kidney or liver tissue lysates, 20 μg protein were separated by 10% sodium dodecyl sulfate-polyacrylamide gel electrophoresis and then transferred to nitrocellulose membranes. The membranes were blocked with 5% milk in Tris buffer solution-Tween 20 (TBS-T) for 1 h at room temperature and incubated overnight at 4°C with a primary antibody in 2% milk in TBS-T. The primary antibodies used in the present study were anti-IGF-1 receptor (IGF-1R, #3027, dilution 1:1,000; Cell Signaling Technology, Danvers, MA, USA), anti-ERK1/2 (SC-94, dilution 1:1,000; Santa Cruz Biotechnology, Santa Cruz, CA, USA), anti-phosphorylated ERK (pERK)1/2 (SC-7383, dilution 1:1,000; Santa Cruz), and anti-glyceraldehyde 3-phosphate dehydrogenase (ab8245, dilution 1:10,000; Abcam, Cambridge, UK). The membranes were then washed 3 times with TBS-T and incubated with a secondary antibody (Santa Cruz) conjugated to horseradish peroxidase with 2% milk in TBS-T. Specific antibody signals were detected with an enhanced chemiluminescence system (ECL or ECL Advance Western Blotting Detection System; Amersham Life Sciences, Arlington Heights, IL, USA, or ImmunoStar LD; Wako). Images of the blots were captured, and the intensity of the protein bands was quantified by CS Analyzer 2.0 with a CCD camera (ATTO Corporation, Tokyo, Japan). The relative band intensity from the kidney of OCT alone-, PAS alone-, and combined OCT and PAS-treated PCK rats was compared with the kidney of CONT-treated PCK rats (set to 100%).

### Immunohistochemistry and immunofluorescence

Sliced paraffin sections were immersed in xylene for deparaffinization, rehydrated to PBS^-^ from ethanol, and then heated in a microwave oven to 100°C for 15 min in 10 mM sodium citrate buffer (pH 6.0) to unmask antigens. The sections were incubated in 0.3% H_2_O_2_/methanol to destroy endogenous peroxidase activity, and blocked with 1% bovine serum albumin and 0.05% NaN_3_ in PBS^-^ for 30 min at room temperature, and then incubated with a primary antibody overnight at 4°C [23, 24]. The primary antibodies used for immunohistochemistry were anti-pERK1/2 (M8159, dilution 1:200; Sigma), anti-pS6 (#4858, dilution 1:100; Cell Signaling), anti-Ki67 (ab16667, dilution 1:100; Abcam). The sections were rinsed and incubated with a biotinylated anti-mouse and anti-rabbit IgG/IgA/IgM secondary antibody (Histofine; Nichirei Biosciences, Tokyo, Japan) for immunohistochemical staining. Immune reaction products were developed with 3,3′-diaminobenzidine. Cells with positive signals were counted in 10 random fields of either kidney or liver sections obtained from 6 rats in each group by a naive observer using a 40× objective. In the kidney, we counted positive cells in both normal and cystic structural areas.

### Statistical analysis

Outcome values are expressed as mean ± SD values. Statistical comparisons were made with Student’s *t*-test or two-way analysis of variance, as appropriate, and differences were considered significant at *P* < 0.05.

## Results

### Effect of somatostatin analogs on kidney weight, cystic index, and renal function

**Fig 1:**
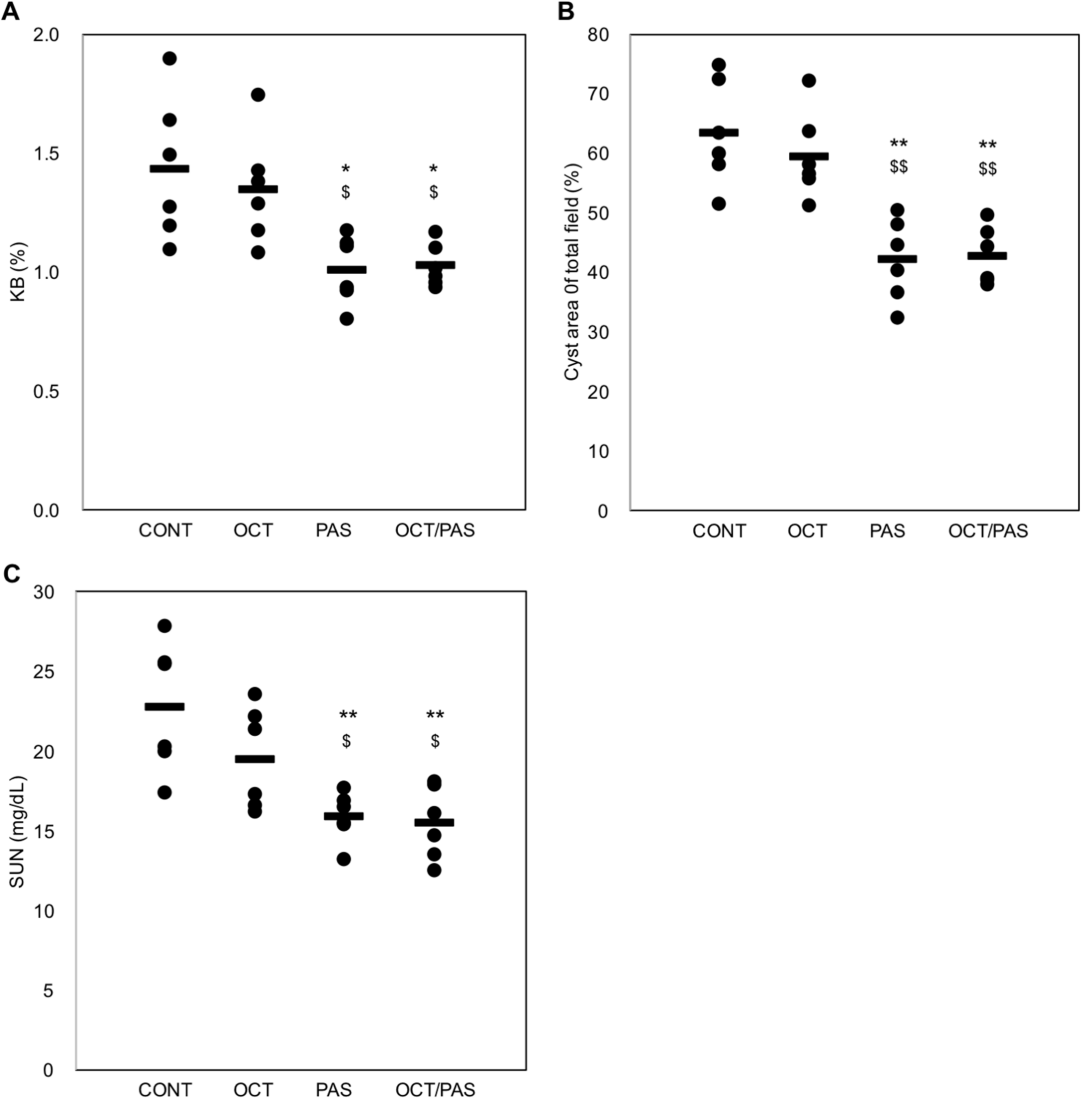
Effects of somatostatin analogs on kidney weight per body weight, cyst area, and renal function. Male PCK rats were assigned randomly to 4 groups (*n* = 6 per each): treated with 8 mg/kg octreotide long-acting release alone (OCT), 8 mg/kg pasireotide long-acting release alone (PAS), co-application of both (OCT/PAS), or vehicle (microparticles liquid; CONT) from 4 to 16 weeks of age. Kidney weight per body weight (KB %, A), cyst area (% of total field, B), and serum urea nitrogen (SUN, mg/dL, C) of individual kidneys are shown by scattered plots. Difference between CONT and each drug-treated group, *: *P* < 0.05, **: *P* < 0.01. Comparison between OCT and PAS or OCT/PAS, $: *P* < 0.05, $ $: *P* < 0.01. There was no statistically significant difference between the PAS and OCT/PAS groups in each measurement.

**Fig 2:**
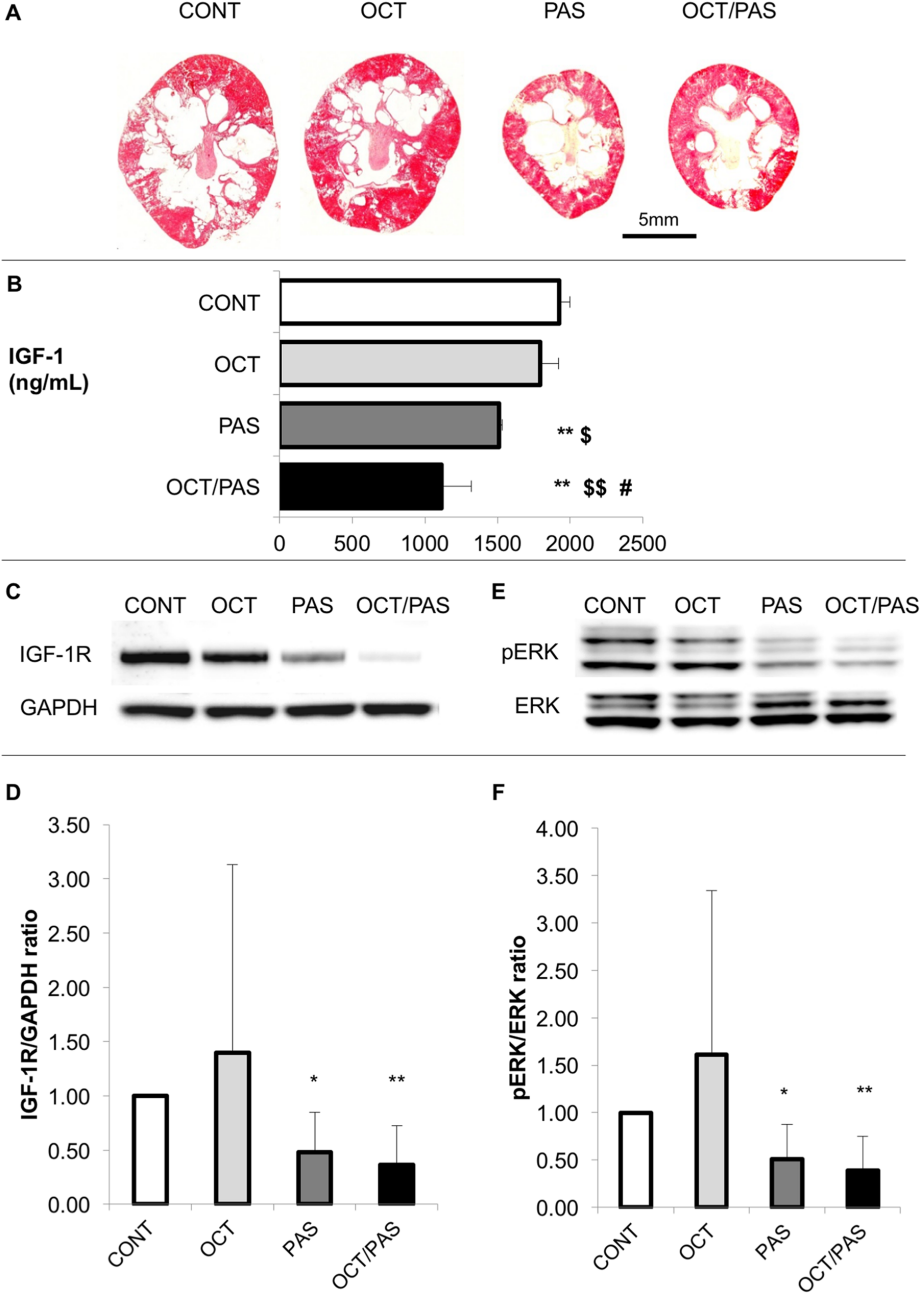
Effects of somatostatin analogs on representative kidney sections, serum IGF-1 levels, renal IGF-1R, and ERK activity. Representative kidney sections were stained with hematoxylin and eosin in each group (*n* = 6 per each, A). Serum insulin-like growth factor-1 (IGF-1) levels (ng/mL, B). The parameters are expressed as mean ± SD. Difference between CONT and each drug-treated group, **: *P* < 0.01. Comparison between OCT and OCT/PAS in PCK males, $: *P* < 0.05, $ $: *P* < 0.01. Comparison between PAS alone and OCT/PAS in PCK males, #: *P* < 0.05. Immunoblots were probed with an antibody to glyceraldehyde 3-phosphate dehydrogenase (GAPDH) or IGF-1 receptor (IGF-1R) (C), and an antibody to extracellular signal-regulated kinase (ERK) or phosphorylated-ERK (pERK) (E). IGF-1R/GAPDH (D) and pERK/ERK (F) ratios were determined from density analysis of the bands. The parameters are expressed as mean ± SD. Difference between CONT and each drug-treated group, *: *P* < 0.05, **: *P* < 0.01.

Food intake was unaffected by OCT, PAS, or their combination (OCT/PAS) (Table 1). Treatment with PAS alone or OCT/PAS reduced body weight and wet kidney weight compared with the vehicle-treated (CONT) group. Percent kidney weight relative to body weight (KB %) was decreased by 41 or 44% in the PAS alone or OCT/PAS group, respectively, compared with CONT (Fig 1A, Fig 2A). However, body weight, wet kidney weight, and KB % were unaffected by treatment with OCT alone. Further, cyst area and SUN level in the rats treated with PAS alone or OCT/PAS were significantly lower compared with CONT (Fig 1B and 1C). Whereas, these were unaffected by treatment with OCT alone. No difference in urine volume and water intake was observed between all groups (S1 Table).

**Table 1:** Effects of somatostatin analogs on food intake, body weight, wet kidney weight.

	CONT	OCT	PAS	OCT/PAS
**Food intake (g/day)**	**21.5 ± 2.6**	**21.3 ± 1.6**	**19.1 ± 5.0**	**19.2 ± 3.4**
**Body weight (g)**	**496 ± 18**	**492 ± 43**	412 ± 42 **, $ $	411 ± 45 **, $ $
**Wet kidney weight (g)**	**7.10 ± 1.64**	**6.59 ± 1.20**	4.13 ± 0.67 **, $ $	4.18 ± 0.50 **, $ $

Male PCK rats were assigned randomly to 4 groups (*n* = 6 per each): treated with 8 mg/kg octreotide long-acting release alone (OCT), 8 mg/kg pasireotide long-acting release alone (PAS), co-application of both (OCT/PAS), or vehicle (microparticles liquid; CONT) from 4 to 16 weeks of age. Food intake (g/day), body weight (g) and wet kidney weight (g) are shown. The parameters are expressed as mean ± SD. Difference between CONT and each drug-treated group

**: *P* < 0.01. Comparison between OCT and PAS or OCT/PAS

$ $: *P* < 0.01. There was no statistically significant difference between the PAS and OCT/PAS groups in each measurement.

Taken together, PAS reduced renal cystic disease progression and helped to maintain renal function either by its sole administration or co-application with OCT.

### Effect of somatostatin analogs on renal cAMP

Since somatostatin analogs are expected to reduce cAMP levels [25], we measured renal tissue cAMP content. cAMP levels were significantly higher in the kidney of PCK rats compared with normal kidney from Sprague Dawley rats in previous study [26]. In the current study, mean ± SD of renal cAMP levels in vehicle-treated PCK rats (*n* = 6) was 2.31 ± 0.62 pmol/mg, whereas it was 1.15 ± 0.08 pmol/mg in age- and gender-matched non-treated Sprague Dawley rats (*n* = 4) in our complementary study. cAMP levels were decreased by PAS and OCT/PAS treatment, but not by OCT alone in PCK rats (Table 2).

**Table 2:** Effect of somatostatin analogs on renal tissue cAMP content.

	CONT	OCT	PAS	OCT/PAS
**Renal cAMP (pmol/mg)**	**2.31 ± 0.62**	**1.86 ± 0.37**	1.63 ± 0.25*	1.56 ± 0.31*

Renal tissue cAMP content was corrected by protein concentration in each sample (*n* = 6 per each group). The parameters are expressed as mean ± SD. Difference between CONT and each drug-treated group

*: *P* < 0.05.

### Effect of somatostatin analogs on serum IGF-1 levels and renal IGF-1R

Mean ± SD of serum IGF-1 levels in vehicle-treated PCK rats (*n* = 6) was 1923 ± 74 ng/mL in the current study. In normal, age- and gender-matched non-treated Sprague Dawley rats (*n* = 4), it was 859 ± 234 ng/mL in the complementary study. Somatostatin analogs are presumed to decrease serum IGF-1 levels by decreasing growth hormone secretion [25]; therefore, we measured serum IGF-1 levels. They were significantly lower in the PAS alone or OCT/PAS group compared with CONT (Fig 2B). Serum IGF-1 levels were lower in the PAS combined with OCT group rather than PAS alone, and were not affected by treatment with OCT alone.

To determine the effect of serum IGF-1 on cellular signaling events, the expression of IGF-1R was measured in kidney specimens. Expression of IGF-1R in the kidney was significantly lower in the PAS alone or OCT/PAS group compared with CONT (Fig 2C and 2D), although no difference was shown between PAS alone and OCT/PAS.

These findings indicate that circulating IGF-1 levels were decreased and the effect of IGF-1 on renal cellular events was reduced by PAS.

### Effect of somatostatin analogs on renal pERK, pS6, and Ki67

**Fig 3:**
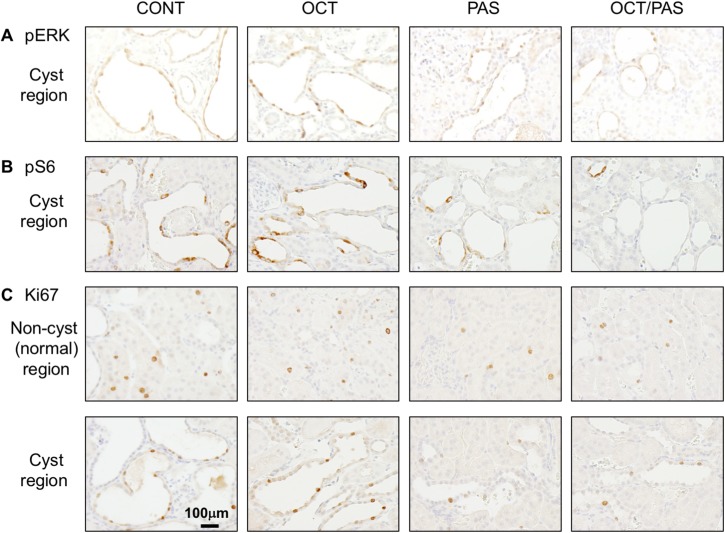
Distribution of pERK-, pS6-, and Ki67-positive cells in kidneys of somatostatin analog-treated PCK rats. Kidney sections were stained with an antibody to pERK (A), pS6 (B), and Ki67 (C), respectively. Nuclei of positive cells are stained as brown with 3,3′-diaminobenzidine, whereas nuclei of negative cells appear blue due to counterstaining with hematoxylin.

Since it is speculated that the expression of IGF-1R has an impact on the activity of the extracellular signal-regulated kinase (ERK) and mammalian target of rapamycin (mTOR) cellular signaling pathways [27], we measured the expression of phosphorylated-ERK (pERK) as an active form of ERK, phosphorylated-S6 ribosomal protein (pS6) as a marker of mTOR activity, and Ki67 as a marker of cell proliferation in kidney specimens. By western blot analyses, the relative band intensity of pERK was significantly lower in the PAS alone or OCT/PAS group compared with CONT (Fig 2E and 2F). No difference was seen between the PAS alone and OCT/PAS groups. By immunohistochemistry staining, the number of pERK-positive cells in renal cystic epithelia was lower in the PAS alone and OCT/PAS groups compared with CONT (Fig 3A, Table 3). No difference was shown between PAS alone and OCT/PAS. The number of pS6-positive cells was significantly lower in the PAS alone and OCT/PAS groups compared with CONT (Fig 3B, Table 3). Further, in the OCT/PAS group, there were a significantly fewer pS6-positive cells than in the PAS alone group. The number of Ki67-positive cells either in cystic and normal tubular epithelia in PAS alone or OCT/PAS was lower compared with CONT (Fig 3C, Table 3). No difference was shown between PAS alone and OCT/PAS.

**Table 3:** Percentage of pERK-, pS6-, and Ki67-positive cells in somatostatin analog-treated kidneys.

	CONT	OCT	PAS	OCT/PAS
**pERK-positive cells (%)**				
**Non-cystic tubules**	**0.13 ± 0.12**	**0.09 ± 0.11**	**0.07 ± 0.09**	**0.05 ± 0.05**
**Cysts**	**11.04 ± 1.12**	**10.99 ± 0.70**	5.14 ± 0.32 **, $ $	4.93 ± 0.78 **, $ $
**Total**	**11.17 ± 1.11**	**11.08 ± 0.71**	5.21 ± 0.33 **, $ $	4.98 ± 0.78 **, $ $
**pS6-positive cells (%)**				
**Non-cystic tubules**	**3.55 ± 1.46**	**3.41 ± 0.66**	**2.40 ± 0.80 $**	**2.32 ± 0.42 $ $**
**Cysts**	**8.01 ± 1.35**	**7.46 ± 0.77**	3.42 ± 0.80 **, $ $	2.85 ± 0.24 **, $ $
**Total**	**11.55 ± 0.89**	**10.88 ± 1.21**	5.82 ± 0.55 **, $ $	5.18 ± 0.19 **, $ $, #
**Ki67-positive cells (%)**				
**Non-cystic tubules**	**4.45 ± 0.98**	**4.71 ± 1.03**	2.14 ± 0.12 **, $ $	2.53 ± 0.32 **, $ $
**Cysts**	**7.15 ± 0.61**	**7.49 ± 1.07**	2.90 ± 0.31 **, $ $	2.78 ± 0.53 **, $ $
**Total**	**11.60 ± 0.95**	**12.20 ± 1.29**	5.04 ± 0.31 **	5.31 ± 0.23 **, $ $

Percentage of pERK-, pS6-, and Ki67-positive cells per total number of cells in cysts and non-cystic tubules in 10 random fields of a kidney section from each rat (*n* = 6 per each group). The Cyst was defined as renal tubules with an inner diameter of 20 μm or more. The parameters are expressed as mean ± SD. Difference between CONT and each drug-treated group

**: *P* < 0.01. Comparison between OCT and PAS or OCT/PAS

$: *P* < 0.05

$ $: *P* < 0.01. Comparison between PAS and OCT/PAS

#: *P* < 0.05.

Taken together, PAS may reduce cell proliferation by inhibiting the ERK and mTOR signaling pathways, and combined treatment with OCT was more robust against mTOR signaling.

### Effects of somatostatin analogs on serum glucose, insulin, glucagon and cortisol levels

Since PAS treatment induces hyperglycemia [19, 20], we measured serum glucose concentration in rats treated with OCT alone, PAS alone, and OCT/PAS (Table 4). Serum glucose levels were significantly higher in the PAS alone group compared with CONT. The elevation of serum glucose by PAS was abolished by its combined treatment with OCT. It is well known that serum glucose concentration is reduced by insulin and increased by several hormones, including glucagon; therefore, we measured serum insulin and glucagon levels (Table 4). Insulin was significantly lower either in OCT, PAS or OCT/PAS group compared with CONT. Glucagon was significantly lower in the OCT alone or OCT/PAS group compared with either CONT or PAS alone.

**Table 4:** Effects of somatostatin analogs on serum glucose, insulin, glucagon and cortisol levels.

	CONT	OCT	PAS	OCT/PAS
**Glucose (mg/dL)**	**116 ± 15**	**126 ± 13**	140 ± 11 *	**118 ± 13 ##**
**Insulin (ng/mL)**	**1.60 ± 0.23**	1.27 ± 0.09 *	1.20 ± 0.20 *	1.24 ± 0.19 *
**Glucagon (ng/mL)**	**0.48 ± 0.12**	0.27 ± 0.11 *	**0.44 ± 0.12 $**	0.17 ± 0.05 **, ##
**Cortisol (ng/mL)**	**73.5 ± 30.6**	**48.5 ± 19.3**	**79.8 ± 38.5**	**61.7 ± 34.8**

Serum glucose (mg/mL), insulin (ng/mL), and glucagon (ng/mL) were measured to determine the underlying mechanism causing the hyperglycemic effect of PAS treatment alone. The parameters are expressed as mean ± SD. Difference between CONT and each drug-treated group

*: *P* < 0.05

**: *P*<0.01. Comparison between OCT and PAS

$: *P* < 0.05. Comparison between PAS and OCT/PAS

##: *P* < 0.01.

These new findings suggest that co-administration of OCT and PAS may avoid the induction of hyperglycemia by PAS by decreasing serum glucagon levels.

### Effect of somatostatin analogs on liver weight, cystic and fibrotic index, and liver function

As PCK rats have hepatic cystic disease with congenital hepatic fibrosis, we also determined the effects of PAS and OCT on the progression of liver disease. Wet liver weight was significantly lower in the PAS alone and OCT/PAS groups than in the CONT group. Percent liver weight relative to body weight (LB %) was significantly lower in the PAS alone group compared with CONT. Cyst area and fibrosis index were significantly lower in the PAS alone and OCT/PAS groups compared with CONT. LB % was unaffected by treatment with OCT alone or in combination with PAS (S1 Fig). AST and ALT levels were in the normal range in all PCK male rats. Neither AST nor ALT was affected by OCT alone, PAS alone, or their combination (S2 Table).

These data indicate that treatment with PAS has an ameliorative effect not only on renal disease progression but also the progression of hepatic disease.

### Effect of somatostatin analogs on cardiovascular function

No difference in HR, DBP, or SBP was seen between all groups at 4 weeks of age (S3 Table). In 15-week-old animals, DBP and SBP were significantly higher in PCK rats compared with Sprague Dawley rats with normal kidneys. No difference in HR was shown between all groups in PCK rats in the current study. DBP was slightly lower (*P* < 0.054) in the PAS alone group and was significantly decreased to the normal range by OCT/PAS combination compared with CONT. SBP was significantly decreased to the normal range by PAS alone and its combination with OCT compared with CONT (S4 Table).

The current results suggest that the reduction of blood pressure by PAS may have a renoprotective effect to ameliorate the progression of kidney disease.

## Discussion and conclusion

The current report has shown that co-administration of OCT and PAS could be a possible choice to ameliorate PKD progression with a reduction of hyperglycemic risk. Previously, Masyuk et al. reported that either OCT or PAS alone has a beneficial effect against disease progression in PCK rats, although PAS was more robust [10, 11]. Hence, we have determined the effect of the co-application of OCT and PAS, i.e., whether their effectiveness is enhanced. In our present study, OCT alone did not ameliorate either renal or hepatic disease in this rat model; whereas, Masyuk et al. showed the reduction of renal and hepatic disease progression by OCT. Although the cause of the different results between their and our study is not clearly addressed, it may because the study-term of drug treatment (from 3 until 9 weeks of age in their study vs. from 4 until 16 weeks of age in our study), and drug form and dose (non-LAR type subcutaneous osmotic pump of 20 [10 or 100] μg/kg/day vs. LAR type subcutaneous injection of 8 mg/kg/28 days). Since LAR-type OCT is being used for the clinical treatment of acromegaly, hypophyseal gigantism, or gastrointestinal hormone-producing tumors, determining its effective dose in rat model and ARPKD patients by a pharmacokinetics study would be an important next step. In our present study, since both insulin and glucagon were significantly lower either in OCT alone or OCT/PAS group compared with CONT or PAS alone, it is suggested that the dosage of OCT was sufficient to reduce PAS-induced glucagon level. Several adverse effects of PAS are known such as diarrhea, weakness, fatigue, nausea, vomiting, low blood pressure, loss of appetite, and altered blood levels of cortisol and glucose. In particular, hypocortisolism and hyperglycemia are noteworthy events. For example, Schmid et al. reported that treatment with PAS alone caused hyperglycemia in rats and healthy humans [20, 25]. It is known that serum glucose concentration is decreased by insulin and increased by glucagon. Our current results suggest that the co-administration of OCT and PAS controls serum glucose by maintaining an appropriate ratio of insulin and glucagon to avoid the risk of an adverse effect. Since OCT has high affinity at SSTR2, activation of this receptor by the current dosage of OCT may be enough to counteract the hyperglycemic effect by reducing glucagon secretion from pancreatic alpha cells [20]. Further, role of IGF-1 pathway in the cystogenesis in renal cystic disease is reported in immortalized epithelial cells from patients and animal models [28, 29]. In the current study, significant body weight reduction was shown in the groups with PAS alone and the co-administration of OCT and PAS in addition to the ameliorative effect against disease progression. In these groups, there was a trend of decreased food intake, although the difference was not statistically significant. Weckbecker et. al. reported that PAS (SOM230) reduced body weight and plasma IGF level of normal rats without a change in food or water intake. They speculate that high affinity for SSTR5 may be crucial for inhibiting growth hormone (GH) release by PAS [20, 30]. Therefore, it is suggested that PAS may have an inhibitory effect on the GH/IGF-1 axis in PCK rats in the present study.

In conclusion, our current study shows two important points. First, the co-administration of OCT and PAS has a robust effect similar to that of PAS alone, both for renal and hepatic disease progression. Second, co-administration with OCT could avoid the hyperglycemia induced by PAS monotherapy, possibly by balancing by the opposing effects of insulin and glucagon. Therefore, it is suggested that co-administration of OCT and PAS may have a potential benefit for ARPKD patients with renal and hepatic disorders to eliminate the risk of the adverse effect of hyperglycemia following sole administration of PAS.

## Supporting information

S1 Fig Effects of somatostatin analogs on representative liver sections. Representative liver sections were stained with hematoxylin and eosin in each group. (A) macrophotographs, (B) microphotographs (×40 magnification).
(TIF)

S1 Table Influences of somatostatin analogs on water intake, urine volume. Water intake (mL/day) and urine volume (mL/day) were measured in 15-week-old PCK rats (*n* = 6). The parameters are expressed as mean ± SD. Difference between OCT and PAS or OCT/PAS, $ $: *P* < 0.01, X: *P* = 0.051.
(DOCX)

S2 Table Effects of somatostatin analogs on liver weight, cyst area, fibrosis index, and liver function markers. Liver weight (LB %) is presented as a percent of total body weight. Cyst area (% of total field) was obtained from representative liver sections stained with hematoxylin and eosin. Fibrosis index (% of total field) was measured from picrosirius red-stained liver sections. Difference between CONT and each drug-treated group, **: *P* < 0.01. Comparison between OCT and PAS or OCT/PAS, $: *P* < 0.05, $ $: *P* < 0.01, X: *P* = 0.052. There was no statistically significant difference between the PAS and OCT/PAS groups in each measurement. Serum aspartate amino transferase (AST, IU/L) and alanine aminotransferase (ALT, IU/L) levels are shown. All parameters are expressed as mean ± SD. There was no statistically significant difference between all groups in each parameter.
(DOCX)

S3 Table Heart rate, diastolic blood pressure and systolic blood pressure in PCK rat groups at 4 weeks of age. Heart rate (HR, bpm), diastolic blood pressure (DBP, mmHg), and systolic blood pressure (SBP, mmHg) were measured in 4-week-old PCK rats (*n* = 6). The parameters are expressed as mean ± SD.
(DOCX)

S4 Table Effects of somatostatin analogs on cardiovascular function. Heart rate (HR, bpm), diastolic blood pressure (DBP, mmHg), and systolic blood pressure (SBP, mmHg) were measured in 15-week-old PCK rats (*n* = 6). The parameters are expressed as mean ± SD. Difference between CONT and each drug-treated group, **: *P* < 0.01, X: *P* = 0.054. Comparison between CONT and PAS, X: *P* = 0.054. Comparison between OCT and PAS or OCT/PAS, $: *P* < 0.05, $ $: *P* < 0.01.
(DOCX)
