# Supplementary figures and images for: Beneficial effect of combined treatment with octreotide and pasireotide in PCK rats, an orthologous model of human autosomal recessive polycystic kidney disease
Source: PLoS One. 2017 May 18;12(5):e0177934. doi: 10.1371/journal.pone.0177934 (PMC5436842; doi:10.1371/journal.pone.0177934)

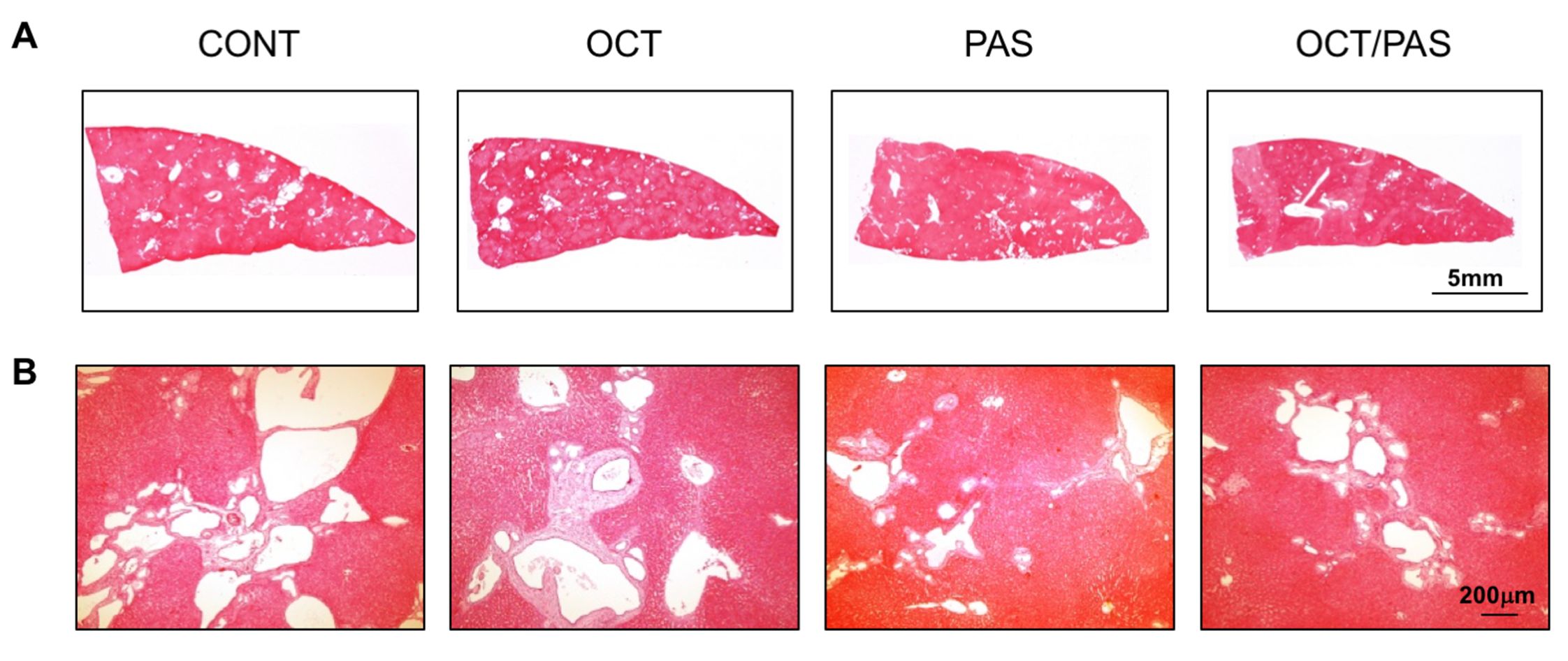

Supplement: S1 Fig — Representative liver sections were stained with hematoxylin and eosin in each group. (A) macrophotographs, (B) microphotographs (×40 magnification). (TIF) [file pone.0177934.s001.tif]
