# Supplementary material for: Beneficial effect of combined treatment with octreotide and pasireotide in PCK rats, an orthologous model of human autosomal recessive polycystic kidney disease
Source: PLoS One. 2017 May 18;12(5):e0177934. doi: 10.1371/journal.pone.0177934 (PMC5436842; doi:10.1371/journal.pone.0177934)
Supplement: S1 Table — Water intake (mL/day) and urine volume (mL/day) were measured in 15-week-old PCK rats (n = 6). The parameters are expressed as mean ± SD. Difference between OCT and PAS or OCT/PAS, $ $: P < 0.01, X: P = 0.051. (DOCX) [file pone.0177934.s002.docx]

|  | **CONT** | **OCT** | **PAS** | **OCT/PAS** |
| --- | --- | --- | --- | --- |
| **Water intake (mL/day)** | **36.3 ± 13.4** | **32.0 ± 13.4** | **32.5 ± 7.0** | **29.8 ± 7.5** |
| **Urine volume (mL/day)** | **20.7 ± 7.8** | **22.3 ± 4.6** | **15.7 ± 5.7 X** | **13.7 ± 4.8 $$** |
