# Supplementary material for: Beneficial effect of combined treatment with octreotide and pasireotide in PCK rats, an orthologous model of human autosomal recessive polycystic kidney disease
Source: PLoS One. 2017 May 18;12(5):e0177934. doi: 10.1371/journal.pone.0177934 (PMC5436842; doi:10.1371/journal.pone.0177934)
Supplement: S2 Table — Liver weight (LB %) is presented as a percent of total body weight. Cyst area (% of total field) was obtained from representative liver sections stained with hematoxylin and eosin. Fibrosis index (% of total field) was measured from picrosirius red-stained liver sections. Difference between CONT and each drug-treated group, **: P < 0.01. Comparison between OCT and PAS or OCT/PAS, $: P < 0.05, $ $: P < 0.01, X: P = 0.052. There was no statistically significant difference between the PAS and OCT/PAS groups in each measurement. Serum aspartate amino transferase (AST, IU/L) and alanine aminotransferase (ALT, IU/L) levels are shown. All parameters are expressed as mean ± SD. There was no statistically significant difference between all groups in each parameter. (DOCX) [file pone.0177934.s003.docx]

|  | **CONT** | **OCT** | **PAS** | **OCT/PAS** |
| --- | --- | --- | --- | --- |
| **Wet liver weight (g)** | **24.4 ± 1.2** | **23.0 ± 2.3** | **17.0 ± 3.4 **, $$** | **16.3 ± 3.3 **, $$** |
| **LB (%)** | **4.92 ± 0.32** | **4.69 ± 0.45** | **4.09 ± 0.48 **, X** | **3.86 ± 0.50 **, $** |
| **Cyst area**  **(% of total field)** | **14.9 ± 2.2** | **13.8 ± 2.8** | **7.4 ± 1.7 **, $$** | **8.7 ± 3.1 **, $** |
| **Fibrosis index**  **(% of total field)** | **13.0 ± 1.5** | **11.3 ± 1.4** | **7.6 ± 2.2 **, $$** | **6.3 ± 1.1 **, $$** |
| **AST (IU/L)** | **91 ± 29** | **74 ± 21** | **84 ± 22** | **88 ± 40** |
| **ALT (IU/L)** | **16.2 ± 3.8** | **16.0 ± 1.2** | **17.0 ± 3.6** | **17.9 ± 2.8** |
