# Supplementary material for: Beneficial effect of combined treatment with octreotide and pasireotide in PCK rats, an orthologous model of human autosomal recessive polycystic kidney disease
Source: PLoS One. 2017 May 18;12(5):e0177934. doi: 10.1371/journal.pone.0177934 (PMC5436842; doi:10.1371/journal.pone.0177934)
Supplement: S3 Table — Heart rate (HR, bpm), diastolic blood pressure (DBP, mmHg), and systolic blood pressure (SBP, mmHg) were measured in 4-week-old PCK rats (n = 6). The parameters are expressed as mean ± SD. (DOCX) [file pone.0177934.s004.docx]

|  | **CONT** | **OCT** | **PAS** | **OCT/PAS** |
| --- | --- | --- | --- | --- |
| **HR (bpm)** | **384 ± 27** | **375 ± 27** | **400 ± 34** | **380 ± 11** |
| **DBP (mmHg)** | **57 ± 4** | **54 ± 8** | **59 ± 3** | **56 ± 5** |
| **SBP (mmHg)** | **97 ± 5** | **94 ± 5** | **93 ± 4** | **96 ± 7** |
