# Supplementary material for: Beneficial effect of combined treatment with octreotide and pasireotide in PCK rats, an orthologous model of human autosomal recessive polycystic kidney disease
Source: PLoS One. 2017 May 18;12(5):e0177934. doi: 10.1371/journal.pone.0177934 (PMC5436842; doi:10.1371/journal.pone.0177934)
Supplement: S4 Table — Heart rate (HR, bpm), diastolic blood pressure (DBP, mmHg), and systolic blood pressure (SBP, mmHg) were measured in 15-week-old PCK rats (n = 6). The parameters are expressed as mean ± SD. Difference between CONT and each drug-treated group, **: P < 0.01, X: P = 0.054. Comparison between CONT and PAS, X: P = 0.054. Comparison between OCT and PAS or OCT/PAS, $: P < 0.05, $ $: P < 0.01. (DOCX) [file pone.0177934.s005.docx]

|  | **CONT** | **OCT** | **PAS** | **OCT/PAS** |
| --- | --- | --- | --- | --- |
| **HR (bpm)** | **332 ± 26** | **329 ± 29** | **318 ± 28** | **326 ± 30** |
| **DBP (mmHg)** | **108 ± 8** | **110 ± 9** | **97 ± 9 X, $** | **96 ± 3 **, $$** |
| **SBP (mmHg)** | **147 ± 2** | **145 ± 3** | **131 ± 4 **, $$** | **128 ± 5 **, $$** |
